# Assessment of MRI issues at 3-Tesla for metallic surgical implants: findings applied to 61 additional skin closure staples and vessel ligation clips

**DOI:** 10.1186/1532-429X-14-3

**Published:** 2012-01-09

**Authors:** Amreeta Gill, Frank G Shellock

**Affiliations:** 1 Biomedical Engineering Interdepartmental Program, University of California, Los Angeles, 420 Westwood Plaza, Engineering IV, 64-144, Los Angeles, CA 90095, USA; 2 Keck School of Medicine, University of Southern California and Institute for Magnetic Resonance Safety, Education, and Research, Los Angeles, CA, USA

**Keywords:** Magnetic Resonance Imaging, Safety, MRI, Implants, Specific Absorption Rate, Artifacts

## Abstract

**Purpose:**

Metallic skin closure staples and vessel ligation clips should be tested at 3-Tesla to characterize MRI issues in order to ensure patient safety. Therefore, metallic surgical implants were assessed at 3-Tesla for magnetic field interactions, MRI-related heating, and artifacts.

**Methods:**

A skin closure staple (Visistat Skin Stapler, staple, Polytetrafluoroethylene, PTFE, coated 316L/316LVM stainless steel; Teleflex Medical, Durham, NC) and a vessel ligation clip (Hemoclip Traditional, stainless steel; Teleflex Medical, Durham, NC) that represented the largest metallic sizes made from materials with the highest magnetic susceptibilities (i.e., based on material information) among 61 other surgical implants (52 metallic implants, 9 nonmetallic implants) underwent evaluation for magnetic field interactions, MRI-related heating, and artifacts using standardized techniques. MRI-related heating was assessed by placing each implant in a gelled-saline-filled phantom with MRI performed using a transmit/receive RF body coil at an MR system reported, whole body averaged SAR of 2.9-W/kg for 15-min. Artifacts were characterized using T1-weighted, SE and GRE pulse sequences.

**Results:**

Each surgical implant showed minor magnetic field interactions (20- and 27-degrees, which is acceptable from a safety consideration). Heating was not substantial (highest temperature change, ≤ 1.6°C). Artifacts may create issues if the area of interest is in the same area or close to the respective surgical implant.

**Conclusions:**

The results demonstrated that it would be acceptable for patients with these metallic surgical implants to undergo MRI at 3-Tesla or less. Because of the materials and dimensions of the surgical implants that underwent testing, these findings pertain to 61 additional similar implants.

## Background

Surgical staples are specialized implants used in surgery in place of sutures and are commonly used to close skin wounds, as well as to connect or remove anatomic areas such as the bowels or lungs [1]. The use of staples is often preferred because it is considered to be faster than suturing by hand and tends to be more accurate and consistent, while creating less tissue trauma [1]. In skin closure, particularly those where aesthetics are not of great concern (e.g., the scalp), the use of skin staples is an increasingly common alternative [1]. Staples are also used in surgery to join tissues, especially to achieve anastomosis of tubular structures including the gastrointestinal tract and vasculature.

As the number of surgical procedures increase, there is a need to develop more efficient techniques and user-friendly tools that address the increasing time constraints and issues of patient satisfaction [2]. One of the issues for vascular procedures has been the labor intensive and time-consuming process of achieving hemostasis [2]. Vascular closure devices have been demonstrated to reduce time to hemostasis and potentially decrease the length of hospital stay [2]. As such, vascular clips have increasingly been used surgically for their hemostatic features.

Skin closure staples and vessel hemostatic or ligation clips are typically made from non-absorbable materials such as stainless steel, cobalt chromium, nitinol, tantalum, titanium or metallic alloys [1,2], although some of these surgical implants may be made from nonmetallic, non-conducting materials and are absorbable. These surgical implants are available in a wide variety of shapes and sizes and selected according to their intended use (e.g., bone, vessel, bowel, lung, or skin) [1,2].

Metallic implants potentially pose hazards or problems for patients referred to magnetic resonance imaging (MRI) [2-11]. To ensure patient safety, *in vitro *test methods are utilized to characterize various MRI issues for a given implant or device [3-12]. Over the years, a variety of staples and vessel ligation clips have been evaluated for magnetic field interactions, heating, and artifacts [3-6,8-11]. While most of these implants were reported to be acceptable for patients undergoing MRI up to and including 3-Tesla or less, some clips deployed endoscopically and made from ferromagnetic stainless steel (e.g., the Resolution Clip, Boston Scientific Corporation) were found to be unsafe for patients [8].

In consideration of the information above, the purpose of our investigation was to assess MRI issues (i.e., magnetic field interactions, MRI-related heating, and artifacts) at 3-Tesla for a skin closure staple and a hemostatic clip.

## Materials and methods

### Surgical clips

**Figure 1:**
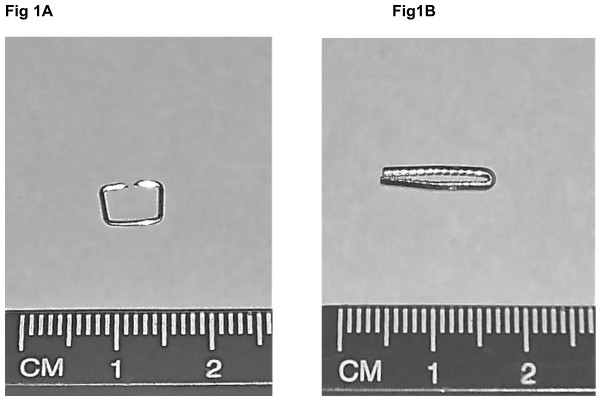
**The skin closure staple**. The skin closure staple (1a) and (1b) hemostatic clip (1b) that underwent testing at 3-Tesla.

The skin closure staple (Visistat Skin Stapler, staple, Polytetrafluoroethylene, PTFE, coated 316L/316LVM stainless steel; Teleflex Medical, Durham, NC) and hemostatic clip (Hemoclip Traditional, stainless steel; Teleflex Medical, Durham, NC) (Figure 1a and 1b) were selected for testing because they represented the largest metallic masses and sizes, and have the highest magnetic susceptibility (i.e., based on material information for all items) among 61 other surgical clips made of metallic (52 clips) or nonmetallic (nine vessel ligation clips) materials. For the metallic surgical implants, these items were made from materials with lower magnetic susceptibility than stainless steel [12] and had smaller dimensions. **Appendix I **presents the details for the surgical clips involved in this investigation. For the nonmetallic clips, the materials were both nonmetallic and non-conducting and, as such, are deemed "MR safe" according to current criteria and labeling terminology [13,14].

### Magnetic field interactions

The metallic surgical implants were evaluated for translational attraction and torque in association with a 3-Tesla MR system (Excite, HDx, Software 14X.M5, General Electric Healthcare, Milwaukee, WI; active-shielded, horizontal field scanner).

#### Translational Attraction

To determine translational attraction for the two different surgical implants, the standardized deflection angle technique was used [6,7,15]. Thus, each implant was connected to a test fixture to measure the deflection angle in the 3-Tesla MR system, at the point of the highest spatial gradient magnetic field [6,7,15,16]. The highest spatial gradient magnetic field for the 3-Tesla scanner used in this investigation is 720 gauss/cm [6,7]. The test fixture incorporated a protractor with 1-degree graduated markings and the implant was suspended on this apparatus by a lightweight string (20-cm in length; weight, less than 1% of the weight of each implant) that was fixed at the 0-degree indicator of the protractor. The maximum deflection angle from the vertical direction to the nearest 1-degree was measured three times for both metallic surgical implants, and an average value was calculated [6,7].

#### Qualitative Assessment of Torque

Torque was determined for the two metallic surgical implants in association with exposure to the 3-Tesla MR system using a previously-described, qualitative assessment technique [6,7]. Each implant was placed on a flat plastic device with a millimeter grid, which was positioned in the center of the 3-Tesla scanner, where the effect of torque is the greatest [6,7]. Each surgical implant was placed on the test apparatus in an orientation that was 45-degrees relative to the static magnetic field and observed for possible alignment or rotation. The implant was then moved 45-degrees relative to its previous position and again observed for alignment or rotation, with this process repeated to encompass a full 360-degrees of rotation [6,7]. A qualitative scale was applied to the findings, as follows [6,7]: 0, no torque; +1, mild or low torque, the implant slightly changed orientation but did not align to the magnetic field; +2, moderate torque, the implant aligned gradually to the magnetic field; +3, strong torque, the implant showed rapid and forceful alignment to the magnetic field; +4, very strong torque, the implant showed very rapid and very forceful alignment to the magnetic field.

### MRI-Related Heating

#### Phantom and Experimental Setup

MRI-related heating at 3-Tesla/128-MHz was assessed for each metallic surgical implant. This procedure used a plastic, ASTM phantom filled to a depth of 10-cm with gelled-saline (i.e., 1.32-g/L NaCl plus 10 g/L polyacrylic acid in distilled water), with each implant placed in a position in the phantom where there was a high uniform electric field tangential to the implant, ensuring extreme RF heating conditions for this experimental set up (i.e., based on an analysis of the ASTM phantom and the MRI conditions used for this assessment) [7,17]. A relatively high level of RF energy was applied during the MRI-related heating experiment, as previously described [7,17].

#### Temperature Recording System and Placement of Thermometry Probes

Temperature measurements were obtained using a fluoroptic thermometry system (Model 3100, LumaSense Technologies, Santa Clara, CA) with fluoroptic thermometry probes (Model SFF-2; 0.5-mm in diameter) positioned on each metallic surgical implant to record representative temperatures, as follows: Probe #1, sensor portion of the probe placed in contact with one end of the implant; Probe #2, sensor portion of the probe placed in contact with opposite end of the implant; Probe #3, sensor portion of the probe placed in contact with middle portion of the implant. The positions of the thermometry probes were inspected and verified immediately before and after each MRI-related heating experiment. In addition, a thermometry probe was placed in the phantom at a position removed (30-cm directly across from the implant, 1-cm from the opposite edge of the phantom) from the implant but within the area of MR imaging, to record a reference temperature during the heating experiment (Probe #4) [7,17].

#### MRI Conditions

MR imaging was conducted at 3-Tesla/128-MHz (Excite, Software G3.0-052B, General Electric Healthcare, Milwaukee, WI), using the body coil to transmit and receive radiofrequency (RF) energy. MRI parameters were selected to generate a relatively high level of RF energy and produced an MR system reported, whole body averaged specific absorption rate (SAR) of 2.9-W/kg for 15-min [7,17]. The land-marking position (i.e., the center position or anatomic region for the MR imaging procedure) and multiple section locations were selected to encompass the entire area of each metallic surgical implant under evaluation (i.e., separate MRI-related heating tests were performed).

#### Experimental Protocol

Each metallic surgical implant was placed in the ASTM phantom at a position mid-line on the left side, slightly (5-mm) below the mid-depth (vertical orientation) of the gelled-saline. For this particular 3-Tesla/128-MHz MR system and experimental set-up, the left side of the ASTM phantom was found to be associated with a greater temperature rise than the right side (i.e., based on pilot experiments). Therefore, each implant was placed on the left side of the ASTM phantom to yield a worst-case temperature rise based on prior analysis of implant heating for this particular MR system (i.e., due to asymmetry in heating patterns for this phantom and MR system) [7,17].

Each metallic surgical implant was positioned in the plastic phantom using a grid and small plastic post set-up, as previously-described [6]. The fluoroptic thermometry system was calibrated and the fluoroptic thermometry probes were applied. The phantom was filled with the gelled-saline and allowed to equilibrate to the environmental temperature for more than 24-hours. The MR system fan was turned off during the MRI-related heating investigation. The room and MR system bore temperatures were at constant levels throughout each experimental session. After recording baseline temperatures (5-min.), MR imaging was performed for 15-min. with temperatures recorded at 5-sec. intervals. This procedure was repeated for the next implant after the gelled-saline returned to thermo-equilibrium facilitated by manual mixing and verified by recording temperatures at multiple positions in the phantom. The highest temperature changes recorded by the fluoroptic thermometry probes are reported for each implant. Using this procedure, the MRI-related heating information applies to a "per pulse sequence" aspect of the MRI examination [7,17].

The "background" temperature was also recorded in the ASTM phantom. Accordingly, the temperature change was recorded at the same position, middle temperature probe position (i.e., corresponding to the position for Probe #3 for the MRI-related heating test with the implant present) in the phantom in association with MRI-related heating of the gelled-saline-filled phantom *without *the implant present. To record the background temperature, a fluoroptic thermometry probe was placed in the ASTM head/torso phantom at a position mid-line on the left side, slightly (5-mm) below the mid-depth (vertical orientation) of the gelled-saline.

### Artifacts

MR imaging artifacts were assessed at 3-Tesla for each the metallic surgical implant. This test was accomplished by performing MR imaging with both implants attached to a plastic frame and then placed in a gadolinium-doped, saline-filled plastic phantom as previously-described [7]. Sufficient distance was placed between the two implants to prevent overlap of the respective artifacts. MRI was performed at 3-Tesla (Excite, HDx, Software 14X.M5, General Electric Healthcare, Milwaukee, WI), using a transmit/receive RF head coil, and the following pulse sequences [7]:

(1) T1-weighted, spin echo pulse sequence; repetition time, 500-msec; echo time, 20-msec; matrix size, 256 × 256; section thickness, 10-mm; field of view, 26-cm; number of excitations, 2; bandwidth; 16 kHz;

(2) Gradient echo (GRE) pulse sequence; repetition time, 100-msec; echo time, 15-msec; flip angle, 30 degrees; matrix size, 256 × 256; section thickness, 10-mm; field of view, 26-cm; number of excitations, 2; bandwidth, 16 kHz.

The imaging planes were oriented to encompass the long axis and short axis of the metallic surgical implants [7]. The frequency encoding direction was parallel to the plane of imaging. Image section locations obtained through the metallic surgical implants were selected from multiple "scout" MR images to represent the largest artifacts for each implant. Planimetry software provided with the MR system was used to measure the cross-sectional areas for the artifacts (i.e., seen as signal loss) associated with the metallic surgical implants. The image display parameters (i.e., window and level settings, magnification, etc) were carefully selected and used in a consistent manner to provide valid measurements of sizes for the artifacts. The accuracy of this measurement method is + 10% [7]. Measurements were obtained to determine the maximum artifact area related to the presence of each implant for each MR imaging condition. This ensured that the sizes of the artifacts for these metallic surgical implants were not underestimated.

## Results

The average deflection angles were 20-degrees for the skin closure staple and 27-degrees for the hemostatic clip. The qualitatively measured torque was 0, no torque in each case. MRI-related heating evaluations for these metallic surgical implants indicated that the highest temperature changes measured by the fluoroptic thermometry probes were equal to or less than 1.6°C, with a background temperature of 1.5°C in each case.

**Figure 2:**
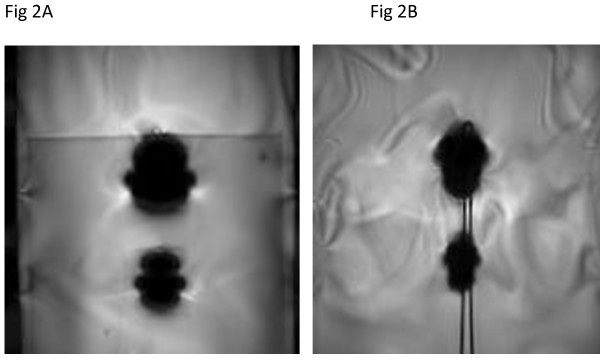
**MRI artifacts**. MRI artifacts associated with the skin closure staple (bottom) and hemostatic clip (top); (a) long axis and (b) short axis imaging planes (GRE pulse sequence; TR/TE 100-msec/15-msec; flip angle, 30 degrees; field of view, 26-cm).

Artifact test results are shown in Table 1. The artifacts were seen as low signal intensity "voids" that were "moderate" in size (i.e., based on a subjective scale of small, moderate, and large) in relation to the size and shape of each metallic surgical implant. The gradient echo pulse sequence produced larger artifacts than the T1-weighted, spin echo pulse sequence. Figure 2 shows examples of artifacts for the skin closure staple and hemostatic clip, as seen on the gradient pulse sequence in the section locations oriented to the long axis (Figure 2(a)) and the short axis (Figure 2(b)) of each device.

**Table 1:** Summary of artifact sizes for the metallic surgical implants evaluated at 3-Tesla.

Skin Closure Clip (Visistat Skin Stapler, Staple)				
Pulse sequence	T1-SE	T1-SE	GRE GRE	
Signal void size (mm^2^)	310	199	648	477
Imaging orientation	long axis	short axis	long axis	short axis
**Hemostatic Clip **(Hemoclip Traditional)				
Pulse sequence	T1-SE	T1-SE	GRE GRE	
Signal void size (mm^2^)	571	364	1,109	877
Imaging orientation	long axis	short axis	long axis	short axis

(Signal void area measured in mm^2^; T1-SE, T1-weighted spin echo; GRE, gradient echo)

## Discussions

### Magnetic Field Interactions

The average deflection angles were 20-degrees for the skin closure staple and 27-degrees for the hemostatic clip, which are acceptable values with regard to translational attraction because the values are less than 45-degrees [6-8,15]. According to the American Society for Testing and Materials International [15], "If the implant deflects less than 45°, then the magnetically induced deflection force is less than the force on the implant due to gravity (its weight). For this condition, it is assumed that any risk imposed by the application of the magnetically induced force is no greater than any risk imposed by normal daily activity in the Earth's gravitational field." The qualitatively measured torque value at 3-Tesla for each metallic surgical implant was 0, no torque. Thus, these implants will not present a risk a patient in the 3-Tesla or less MRI environment with regard to magnetic field interactions (translational attraction and torque). Additional consideration may be given to the "intended use" of these metallic surgical implants insofar as the closing forces that are present will further prevent potential concerns with regard to movement or dislodgement. Indeed, the closing forces of the clips will further mitigate and reduce concerns of the measured, minor magnetic field interactions of these implants.

In general, the factors that impact magnetic field interactions for an implant or device often found in patients referred for MRI procedures include the strength of the static magnetic field, the maximum spatial gradient magnetic field, the dimensions and shape of the object, and the magnetic susceptibility of the material(s) used to construct the object [3-9,15,16]. The skin closure staple and hemostatic clip that were selected for this investigation represented the largest versions with regard to their dimensions and the ones with the highest magnetic susceptibility values for the materials. Therefore, the findings for magnetic field interactions can be appropriately applied to the additional skin closure staples and hemostatic clips presented in **Appendix I **because they have the same or smaller dimensions and are made from materials with lower magnetic susceptibilities [7,12].

### MRI-Related Heating

The results of the MRI-related heating experiments using an MR system reported, whole body averaged SAR (2.9-W/kg) indicated that the highest temperature changes for these metallic surgical implants were less than or equal to 1.6°C. The maximum temperature level for these implants should be considered in reference to the recorded background temperature (i.e., without the implant present in the phantom) for these same MRI conditions, which was 1.5°C. Therefore, the contribution of each implant was only 0.1°C. Notably, the minor temperature rise of 1.6°C will not cause a thermal injury in a human subject.

High increases in temperatures during MRI have been reported for various metallic implants but only occurs in association with an object that has a certain length and/or is in the shape of a closed-loop with a relatively large diameter [8,9]. For the small metallic surgical implants involved in this study, the maximum dimensions and "closed-loop" (i.e., when applied to tissue) aspects are minimal and, therefore, these factors will not be responsible for generating excessive heating during an MRI examination. Because the metallic surgical clips that underwent testing had the largest or similar dimensions compared to those shown in Additional File 1, the findings from the MRI-related heating experiments can be applied to these other surgical implants, with assumed temperature increases that are comparable to those observed in this investigation. Similar results were reported in the evaluation of intracranial aneurysm clips [7]. Of further support for the lack of substantial temperature rises in small metallic implants, the ASTM International document clearly states the following [17]: "Simple metallic structures less than 2-cm in dimension are not expected to exhibit clinically significant RF-induced temperature rise." Importantly, since none of the surgical implants presented herein have dimensions that exceed a length of 2-cm, there is no concern of MRI-related heating related to the conditions used at 3-Tesla or less.

### Artifacts

Artifacts associated with these metallic surgical implants made from stainless steel were categorized as "moderate" in size in reference to their dimensions. Thus, while it is possible that the artifacts may present problems if the MR imaging area of interest is in or near the area where the respective implant is located, pulse sequence optimization techniques commonly used when metal objects are present can substantially mitigate the impact of the artifacts on the diagnostic use of MRI. Because the magnetic susceptibility of the material used for a given implant is the predominant factor responsible for the size of the artifact [7-12], the artifacts associated with the other surgical implants (Additional File 1) are anticipated to be the same size or smaller due to the similar or smaller dimensions and the use of materials with lower magnetic susceptibilities (e.g., lower values for tantalum, titanium, and nonmetallic materials). Regardless, artifacts observed on MR images are not considered to pose safety issues.

#### Conclusions and MRI recommendations

Because of the lack of substantial magnetic field interactions (translational attraction and torque) and minor temperature rises above the background heating during the use of a relatively high MR system reported, whole body averaged SAR, along with the characterization of artifacts, the skin closure staple (Visistat Skin Stapler, Teleflex Medical, Durham, NC) and hemostatic clip (Hemoclip Traditional, Teleflex Medical, Durham, NC) are "MR conditional" using the current criteria applied to MRI evaluations and labeling for implants and devices [7,13,14]. Full labeling for each surgical implant includes, the following information based on the methodology used for testing [13,14,16]:

##### Static magnetic field

-Static magnetic field of 3-Tesla or less

-Maximum spatial gradient magnetic field of 720-Gauss/cm or less

##### MRI-related heating

In non-clinical testing, the surgical implant produced the following temperature rise during MRI performed for 15-min of scanning (i.e., per pulse sequence) in the 3-Tesla (3-Tesla/128-MHz, Excite, HDx, Software 14X.M5, General Electric Healthcare, Milwaukee, WI) MR system: *Highest temperature change*, 1.6°C. Therefore, the MRI-related heating experiments for this surgical implant at 3-Tesla using a transmit/receive RF body coil at an MR system reported whole body averaged SAR of 2.9 -W/kg indicated that the greatest amount of heating that occurred in association with these specific conditions was equal to or less than 1.6°C.

##### Artifact information

MR image quality may be compromised if the area of interest is in the exact same area or close to the position of the surgical implant. Therefore, optimization of MR imaging parameters to compensate for the presence of this device may be necessary.

#### Implications for other metallic and nonmetallic surgical implants

Importantly, the "MR conditional" findings for the two metallic surgical implants can be applied to 52 additional surgical implants (Additional File 1) that have the same or smaller dimensions and made from materials with lower magnetic susceptibilities. This strategy was successfully used in a previous MRI evaluation of aneurysm clips whereby three aneurysm clips underwent testing and the resulting information was applied to many other similar clips [7]. In fact, the U.S. Food and Drug Administration accepted the MRI test results obtained from the three clips that represented the largest versions with regard to their dimensions and the ones with the highest magnetic susceptibility values for the materials, as being appropriate to apply to MR conditional labeling for 155 additional aneurysm clips [7].

Nine clips used for vessel ligation and made from nonmetallic, non-conducting materials (i.e., acetal homopolymer or ticona nylon) are included in Additional File 1 because MRI healthcare professionals may be unaware that these implants exist and, more importantly, may not be familiar with the fact that these clips are considered "MR safe" according to the current criteria and labeling terminology (i.e., MR safe is an item that poses no known hazards in all MRI environments. Using the terminology, "MR Safe" items are non-conducting, non-metallic, and non-magnetic items)[13,14].

## Conclusions

The results from this investigation demonstrated that it would be acceptable for patients with these particular metallic surgical implants to undergo MRI at 3-Tesla or less. Because of the materials and dimensions of the surgical implants that underwent testing, the findings pertain to 61 additional similar implants, thus, effectively expanding the list of implants deemed acceptable for patients undergoing MRI under the conditions used for this study.

## Abbreviations

SAR: specific absorption rate; T1-SE: T1-weighted spin-echo; GRE: Gradient echo.

## Competing interests

The authors declare that they have no competing interests.

## Authors' contributions

AG and FGS, together, made substantial contributions to the conception and design of this study, the acquisition of the data, the analysis and interpretation of data, the drafting of the manuscript and revising it critically for important intellectual content, and gave final approval of the version to be published.

## Supplementary Material

Additional file 1 **Appendix 1**. In consideration of the materials and sizes associated with the skin closure staple (Visistat Skin Stapler, staple, Polytetrafluoroethylene, PTFE, coated 316L/316LVM stainless steel; Teleflex Medical, Durham, NC) and vessel ligation clip (Hemoclip Traditional, stainless steel; Teleflex Medical, Durham, NC) that underwent MRI testing for magnetic field interactions, MRI-related heating, and artifacts at 3-Tesla, the findings from this investigation pertain to those surgical implants listed in **Appendix 1**. *Denotes the particular surgical implant that underwent MRI testing. These surgical implants are listed alphabetically in "MR conditional" and "MR safe" categories. Note that nine surgical implants made from nonmetallic, non-conducting materials are "MR safe" according to current criteria and labeling terminology [13,14]. Click here for file
